# Potential action of androstenedione on the proliferation and apoptosis of stromal endometrial cells

**DOI:** 10.1186/1477-7827-2-81

**Published:** 2004-12-10

**Authors:** Manuel A Maliqueo, Susana Quezada, Marisa Clementi, Ketty Bacallao, Mabel Anido, Cecilia Johnson, Margarita Vega

**Affiliations:** 1 Institute of Maternal and Child Research, School of Medicine, University of Chile, Santiago, Chile; 2 Laboratory of Endocrinology and Metabolism, Department of Internal Medicine, School of Medicine, University of Chile, Santiago, Chile

## Abstract

**Background:**

Hyperandrogenic conditions have been associated with a high prevalence of endometrial pathologies related to cell survival. However, the action of androgens on proliferation and apoptosis in endometrial cells is poorly understood. Therefore, the aim of the present study was to evaluate the effect of androstenedione on cell proliferation, cell death and expression of estrogen receptor (ER) isoforms and proteins related to apoptosis in endometrial cells using two in vitro experimental approaches.

**Methods:**

The endometrial tissue was obtained from 20 eumenorrheic women [28.7 (25 – 35) years] during the early secretory phase. We analyzed cell proliferation (immunohistochemistry of Ki-67 and spectrophotometric assay); apoptosis (DNA fragmentation (TUNEL) and Annexin V-FITC binding); ER-alpha, ER-beta bcl-2 and bax mRNA abundance (RT-PCR) in explants and isolated endometrial epithelial (EEC) and stromal cells (ESC) incubated with androstenedione 1 micro mol/l (A4) or A4 plus hydroxyflutamide 10 micro mol/l (F) for 24 h.

**Results:**

In explants, A4 induced an increase of cell proliferation and a decrease on apoptosis in the stromal compartment (p < 0.05). In isolated ESC, proliferation augmented with A4 (p < 0.05), whereas, no significant modifications in the expression of ER-alpha, ER-beta bcl-2 and bax nor in the apoptotic index were observed. In EEC, A4 increase the ER-beta mRNA abundance (p < 0.05) and a decrease of the bcl-2/bax ratio (p < 0.05), without an increase in the apoptotic index. Hydroxyflutamide reverted the effect of androstenedione on the parameters described.

**Conclusions:**

These results indicate that androstenedione may modulate cell survival, expression of ER-beta and proteins related to apoptosis, suggesting a potential mechanism that associates the effect of hyperandrogenemia on the endometrial tissue.

## Background

Polycystic ovary syndrome (PCOS) is a complex endocrine-metabolic disorder, associated to hyperandrogenism, menstrual disturbances and in many cases to insulin resistance [1,2]. It has been observed that in some of PCOS women, the endometrium is thicker than that of normal cycling women [3] and a higher prevalence of endometrial hyperplasia and adenocarcinoma have also been described in these women [4-6]. The latter may indicate that the mechanisms that regulate the process of cell survival may be disrupted in the endometrium of PCOS women. Recently, we have shown that the expression of proteins involved in the regulation of apoptosis in PCOS endometria were altered [7]. Besides, we and other investigators demonstrated an elevated expression of the estrogen receptor (ER) and its co-activators in endometria of women bearing this syndrome [7-9]. Nevertheless, in those studies it was difficult to establish the exact contribution of androgens as a regulatory steroid of endometria of PCOS women, since multiple factors could be affecting their endometrial function, including hyperinsulinemia and the possible contribution of the unopposed effect of estrogens [10,11]. Therefore, in vitro experimental models such as tissue and cell culture may constitute interesting approaches to determine the potential role of androgens in the regulation of endometrial cell survival.

Early reports in isolated endometrial stromal cells (ESC) have shown that androgens can induce decidualization and inhibition of the expression of ER and progesterone receptors [12,13]. Moreover, in endometrial epithelial cells (EEC), androgens altered the expression of proteins related to uterine receptivity [8] and induced a decrease in the proliferation capacity of those cells [14].

Cell proliferation and apoptosis of the endometrium are importantly regulated by the expression of ER [15], which exists in two major subtypes, estrogen receptors alpha (ERα) and estrogen receptor beta (ERβ). The two isoforms of ER derive from separate genes, with different ligand binding affinities and the response of the tissue to estrogens will depend upon their relative concentrations [16].

On the other hand, in some tissues including the human endometrium, the control of apoptosis has been associated to proteins related to the Bcl-2 family, like Bcl-2 that promotes cell survival and Bax which is an inducer of apoptosis [17,18]. Evenmore, other proteins are involved in the machinery of cell death like caspases which are associated with the cleavage and thus, breakdown of cell structure [19]. In regard to this issue, we have demonstrated that the expression of bcl-2 and bax is increased in the stromal compartment of the endometrium of PCOS women, but we could not observe an increase in the apoptotic index in the endometria of these women [7].

Previous reports have shown that androstenedione is an important androgen detected in the endometrial tissue [20,21]. Therefore, based on the amount of androgens normally found in endometria and their potential importance in alterations of the endometrial cell survival in PCOS women, the objective of the present study was to evaluate the effect of androstenedione on cell proliferation, apoptosis and the expression of ER isoforms and proteins related to apoptosis using two in vitro experimental approaches.

## Methods

### Subjects

Endometrial tissue was obtained with pipelle suction curette from the corpus of the uteri of 20 women with regular menstrual cycles, aged 28.7 (25 – 35) years, at the time of bilateral tubal ligation at the San Borja-Arriarán Clinical Hospital, National Health Service, Santiago, Chile. The tissue was obtained during the early secretory phase since the cells obtained from this phase maintain a high degree of proliferation capacity [22]. None of these women had taken oral contraceptives or other medications for at least 6 months before starting the study. Women who had evidences of PCOS, endometriosis and/or endometrial hyperplasia were excluded. This investigation was approved by the Institutional Ethics Committee of the San Borja-Arriarán Clinical Hospital and an informed written consent was obtained from all subjects.

### Culture System

#### Explants

Human endometrium was cut into slices (20 to 50 mg wet weight) and incubated in 1 mL of Hank's media supplemented with 2 mmol/L glutamax-I (BRL, Life Technology, Bethesda, MD, USA), insulin-transferrin-selenium (ITS) solution (BRL), 0.1% w/v bovine serum albumin (BSA; Sigma Chemical Co., St Louis, MO, USA), 26 mmol/L of NaHCO_3_, 25 mmol/L of HEPES aminoacids solution, 100 IU/ml of penicillin, and 5 mg/mL of streptomycin (Sigma). Incubation was performed during 6 h at 37°C in 5% CO_2_/air in humidified atmosphere in the absence or presence of androstenedione 10^-7 ^to 10^-5 ^M (Sigma) or androstenedione 10^-6 ^M plus hydroxyflutamide 10^-5 ^M (Sigma), the latter added 30 min before androstenedione. After incubation, one piece from both basal and treated tissue explants were frozen in liquid N_2 _and maintained at -70°C for RT-PCR protocols. Another piece was fixed in 4% buffered formaldehyde for 24 h, embedded in paraffin, and cut into 5 μm thick sections before in situ analysis of apoptosis and immunohistochemistry.

#### Cells

The cells were separated and purified according to previously described methods [23]. Briefly, the tissue was cut into small pieces and suspended in Dulbecco's modified Eagle medium (DMEM) (GibcoBRL), collagenase (370 IU/100 mg tissue) (Worthington, Biochemical Corp. Freehold, NJ, USA) and DNAse (14 KU/100 mg tissue) (Sigma) during 1 h at 37°C. Epithelial cells, predominantly from glands, were separated from ESC by decantation and the supernatant containing the ESC was filtered, centrifuged and the cell pellet washed in DMEM. Stromal cells were incubated in appropriate cell culture media (ESC media) (DMEM/MCDB-105 (3:1 v/v), 2% charcoal stripped fetal bovine serum (FBS) (GibcoBRL), insulin-transferrin-selenium (ITS) solution, 2 mmol/L glutamax-I (GibcoBRL), 0.25 μg/mL ascorbic acid (GibcoBRL), 0.25 μg/mL fungizone (GibcoBRL), 100 IU/mL penicillin and 5 mg/mL streptomycin at 37°C in 5% CO_2_/air in humidified atmosphere until confluence.

The glands cells were washed in DMEM and incubated for 1 h (30 min in each side of the culture flask), and then cultured in EEC culture media (DMEM/MCDB-105 (3:1, v/v), 10% charcoal stripped FBS, 2 mmol/L glutamax-I, 0.25 μg/mL ascorbic acid, 0.5 mg% insulin (Sigma), 1 μg% transferrin (Sigma), antibiotic and fungizone), similarly to ESC. In both cell cultures, the media were changed every 3 days. Upon reaching confluence, ESC and EEC were passaged by treatment with 0.5 g/L tripsin-0.2 g/L EDTA solution (GibcoBRL).

The purity of cell cultures was greater than 90% for ESC and EEC, evaluated by immunocytochemistry of vimentin and cytokeratin, respectively.

### Epithelial and stromal cell culture

Cells, EEC and ESC, were grown in appropriate medium in 6-well or 96-well plates at 2.5 × 10^5 ^cells/well or 0.1 × 10^5 ^cells/well, respectively. When cells were subconfluent (48 – 72 h of culture), the media were changed to Hank's media and incubated for 24 h. Then, the cells were incubated in fresh Hank's media at 37°C in 5% CO_2_/air in humidified atmosphere in the absence or presence of androstenedione 10^-6 ^M or androstenedione 10^-6 ^M plus hydroxyflutamide 10^-5 ^M. The latter was added 15 min before androstenedione. The culture was carried out for 24 h to evaluate the early effect of androstenedione on cell survival. The concentration of androstenedione used in the present investigation was established in dose-response experiments and are in agreement with those previously reported [14].

### Immunohistochemical detection

Sections (4 to 6 μm) of human endometrial tissue obtained from cultured explants were deparaffinized in xylene and hydrated gradually through graded alcohols. Endogenous peroxidase activity was prevented by incubating the samples in 3% hydrogen peroxide for 5 min. The sections were incubated in 10 mM sodium citrate buffer (pH 6.0) at 95°C for 20 min. Nonspecific antibody binding was prevented with 2% PBS-BSA for 1 h. Primary antibody of Ki-67 (1:200; Novocastra Laboratories, Newcastle, UK) was applied to the samples and incubated overnight at 4°C; the antibody for caspase-3 (1: 100; R&D System, Inc., Minneapolis, MN) was incubated for 1 h at 37°C and the antibody for androgen receptor (AR) was incubated overnight at 4°C (1: 75; Santa Cruz, CA, USA). The second antibody used in both cases was a biotinylated rabbit antimouse immunoglobulin. The reaction was developed by the streptavidin-peroxidase system and 3,3'diaminobenzidine was used as the chromogen; counterstaining was carried out with hematoxylin. The slides were evaluated in a Nikon optical microscope (Nikon Inc., Melville, NY, USA). The immunohistochemical evaluation was determined as the percentage of positive stained cells. In all cases, at least 500 cells were evaluated by three independent observers.

### In Situ 3'-End Labeling of DNA in Apoptotic cells (TUNEL)

Programmed cell death was detected using TdT-mediated dUTP nick end labeling as previously described (Promega, Madison, WI, USA) [18]. Briefly, paraffin sections were dewaxed with xylene and rehydrated for 3'-end labeling. Tissue sections were incubated with proteinase K (20 μg/ml) at room temperature for 10 min and washed with PBS for 5 min. Then, incubated for 1 h at 37°C with the nucleotide mix labelled with fluorescein and terminal deoxynucleotidyl transferase enzyme and counterstained with propidium iodide. The number of apoptotic cells was quantified by at least counting 1000 cells in a fluorescence microscope by three independent observers. The number of apoptotic cells was expressed as the percentage of positive cells.

### Cell proliferation

CellTiter 96 AQueous Cell Proliferation Assay (Promega) was used to perform cell proliferation, following manufacturer's instructions. Briefly, EEC or ESC were plated in 96-well until sub-confluence; then, the cells were cultured with androstendione 10^-6 ^M alone or androstenedione 10^-6 ^M with hydroxyflutamide 10^-5 ^M, as described above. Twenty μL of a mix of tetrazolium compound (3-(4,5-dimethylthiazol-2-yl)-5-(3-carboxymethoxyphenyl)-2-(4-sulfophenyl) 2H-tetrazolium; MTS) and an electron coupling reagent (phenazine methosulfate; PMS) were added to each well and incubated for 2 h at 37°C. The quantity of the soluble formazan product was measured at 450-nm absorbance in an ELISA reader (Sigma) and expressed as optical density units (OD).

### Detection of apoptosis by Annexin V

Apoptosis was performed using Annexin V-FITC Apoptosis Detection Kit (Oncogene Reasearch Products, Boston, MA, USA). Briefly, after EEC and ESC achieved sub-confluence in 6-well plates, the cells were incubated with androstenedione 10^-6 ^M alone or androstenedione 10^-6 ^M with hydroxyflutamide 10^-5 ^M, as described above. The cells were dissociated by 0.25 g/L trypsin-0.1 g/L EDTA treatment, gently re-suspended in cold binding buffer to approximately 1 × 10^6 ^cells, and incubated with Annexin V-FITC, as indicated by the manufacturer. The cells were counter-stained with propidium iodide, analyzed and counted by two independent observers in at least 1000 cells in each experimental condition using a fluorescent and optical microscope (Nikon, UFX-NX model. Nikon Inc., Melville, NY, USA). The results are expressed as percentage of apoptotic Annexin V-FITC positive cells respect to total cells counted.

### RNA isolation and semiquantitative Reverse Transcription-Polymerase Chain Reaction

Total RNA was isolated using TRIzol Reagent (GibcoBRL) from endometrial tissue and ESC culture according to the manufacturer's instructions. Total RNA was then reverse transcribed, and cDNA was subjected to polymerase chain reaction (PCR) using specific primers for ERα and ERβ[24], bcl-2 and bax cDNA (NIDig 4558699) [7,18]. β-actin was used as an internal control.

Semiquantitative RT-PCRs were achieved in the exponential linear zone amplification for each gene studied. Two μg of total RNA were used for reverse transcription in a total volume of 20 μl using the Revertaid H Minus M-Mulv Reverse (Fermentas Hannover, MD, USA). The PCR condition for ERα and ERβ was 2 mM MgCl_2_, 0.15 mM of dNTP, 0.63 U of Taq DNA polymerase (Fermentas Hannover, MD, USA), and 0.4 μM of each primer; for bcl-2 and bax was 3 mM MgCl_2_, 0.25 mM of dNTP, 0.63 U of Taq DNA polymerase, and 0.4 μM of each primer. The PCR amplification was carried out in the Thermocycler, model PTC-100 (MJ Research Inc, Watertown, MA), as previously reported [23].

The PCR products were electrophoretically resolved on 1% agarose gel and stained with ethidium bromide. The bands were evaluated using an image analyser (Kodak Electrophoresis Documentation and Analysis System [EDAS 290] and Kodak 1D Image Analysis software, Rochester, NY, USA), and normalized relative to β-actin PCR product and expressed as arbitrary units (AU).

To confirm the specificity of RT-PCR products, the fragments were purified with CONCERT Rapid PCR Purification System (GibcoBRL) and sequenced using an ABI PRISM310 automated Sequencer (Perkin Elmer; Norwalk, CT, USA).

### Statistical evaluation

The results are expressed as the percentage of changes obtained in the treated respect to the basal condition. Comparisons between groups were performed by ANOVA following Dunnett test. The significance level was set at 5%. Results are expressed as mean ± standard error of the mean (SEM).

## Results

### Explants culture

**Figure 1:**
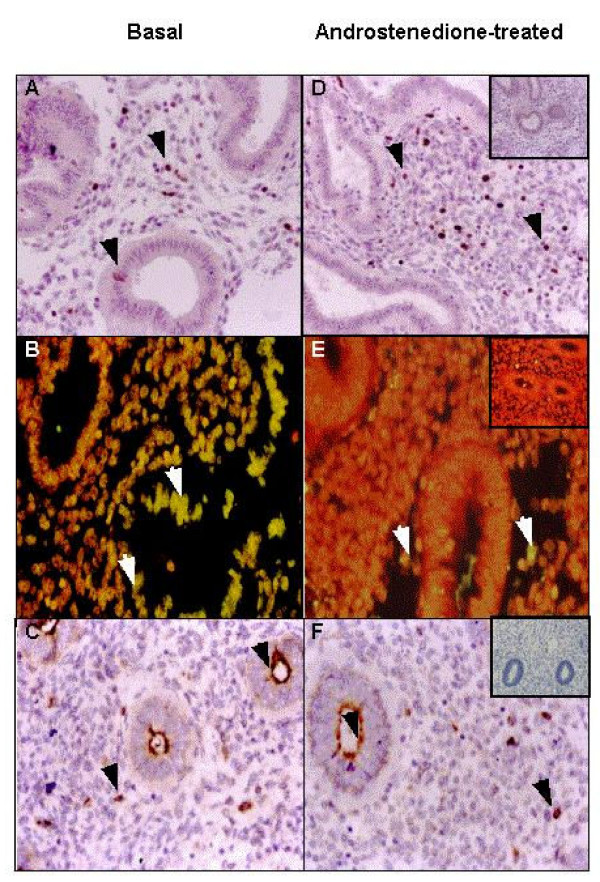
Effect of androstendione on endometrial cell proliferation and apoptosis of human endometria. Basal condition (left panel) and androstenedione-treated explants (right panel). The nucleolar antigen Ki-67, evaluated by immunohistochemistry, was detected in the nucleus of both cell compartments (A, D), indicative of cell proliferation. The nucleus of positive cells for TUNEL, determined by TdT-mediated dUTP nick end labeling, were stained in yellow and counterstained with propidium iodide (B, E), showing DNA fragmentation. The positive staining for caspase-3, determined by immunohistochemistry in paraffin wax sections of endometria, was found in the cell cytoplasm of both compartments (C, G). Negative controls (inserts) for inmunohistochemistry was performed with non-immune rabbit serum in the place of the respective primary antibody and for TUNEL, by the replacement of TdT enzyme. Arrowheads indicate positive staining of the respective proteins. Magnification in all panels, ×400.

In all samples, the expression of the AR was present in the nucleus of EEC and ESC cells (data not shown). Moreover, in EEC showed a positive staining in the cytoplasm. The nucleolar antigen Ki-67 was detected in the nucleus of EEC and ESC. After six hours of incubation with androstenedione at different concentrations (10^-7 ^M – 10^-5 ^M), the percentage of positive cells increased significantly only in the presence of androstenedione 10^-6 ^M and on the stromal compartment (basal: 9,9 ± 2.5%; treated: 21.2 ± 6.4%; p < 0.05) (Figures 1A and 1D). Therefore, the following experiments were performed at a concentration of androstenedione 10^-6 ^M.

On the other hand, TUNEL positive cells were lower in ESC after androstenedione treatment (basal: 21.2 ± 5.0%; treated:10.3 ± 3.7%; p < 0.05), (Figures 1B and 1E). The expression of caspase-3 did not change with the androstenedione treatment in both compartments (Figures 1C and 1F). The addition of hydroxyflutamide plus androstenedione did not modify the degree of proliferation or cell death in tissue explants.

### Isolated endometrial cells

Basal values of cell proliferation were 0.39 ± 0.09 OD and 0.64 ± 0.16 OD for EEC and ESC, respectively, whereas, the percentage of annexin V positive cells was 16.3 ± 4.6% for EEC and 12.7 ± 4.3% for ESC. The addition of androstenedione significantly increased cell proliferation in ESC cultures (Table 1; p < 0.05), and this effect was reverted by the addition of hydroxyflutamide (0.63 ± 0.16 OD vs 0.70 ± 0.19 OD); no changes were observed in the EEC subpopulation (Table 1).

**Table 1:** Effect of androstenedione in cell proliferation and apoptotic index in endometrial epithelial cells (EEC) and endometrial stromal cells (ESC) in vitro.

	Cell Proliferation (%)		Apoptotic index (%)	
	EEC	ESC	EEC	ESC

Basal	100	100	100	100
Androstenedione (10^-6 ^M)	91.6 ± 6.9	135.4 ± 1.2*	165.0 ± 59.0	120.0 ± 11.0
Androstenedione (10^-6 ^M) plus hydroxyflutamide (10^-5 ^M)	99.1 ± 10.0	109.2 ± 4.2	111.2 ± 6.3	106.7 ± 7.4

*p < 0.05. Values are calculated as percentage of basal and are expressed as mean ± SEM.

The percentage of cells with positive signs of apoptosis was similar between the basal and the treated conditions, independently of the cell type analyzed. The addition of hydroxyflutamide plus androstenedione to both cell cultures did not modify the degree of cell death (Table 1).

### Effect of androstenedione on the abundance of messenger RNA for bcl-2 and bax

A similar mRNA abundance for bcl-2 and bax was obtained in tissue explants without treatment (bcl-2; 0.96 ± 0.12 AU; bax: 0.99 ± 0.17 AU). Table 2 shows the effect of androstenedione on the mRNA abundance of bcl-2 and bax in endometrial explants. For bcl-2, the level of its mRNA decreased with androstenedione treatment (p < 0.05) and hydroxyflutamide inhibited this effect, whereas, the mRNA abundance of bax did not change with the treatment. Despite these results, a similar bcl-2/bax ratio was obtained (basal: 1.06 ± 0.21; treated: 0.99 ± 0.12).

**Table 2:** Effect of androstenedione in the mRNA abundance for bcl-2 and bax in endometrial tissue explants.

	bcl-2 (%)	bax (%)
Basal	100	100
Androstenedione (10^-6 ^M)	73.6 ± 3.9*	90.0 ± 20.9
Androstenedione (10^-6 ^M) plus hydroxyflutamide (10^-5 ^M)	86.7 ± 14.5	113.3 ± 37.1

*p < 0.05. Values are calculated as percentage of basal and are expressed as mean ± SEM.

**Figure 2:**
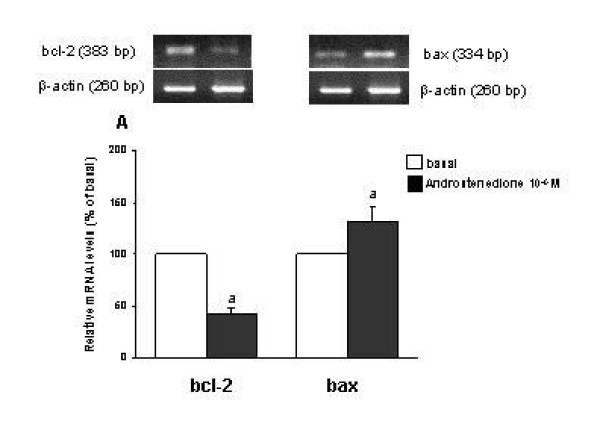
Polymerase chain reaction (PCR) amplification from reverse-transcribed cDNA from endometrial epithelial cells (EEC) under the stimulation with androstenedione 10^-6 ^M, using primers for bcl-2, bax and β-actin. Results represent six experiments performed in duplicate. Normalized yield for bcl-2 and bax PCR fragments relative to β-actin. PCR products from different experiments are shown as percentage respect to basal. The values are expressed as mean ± SEM. ^a^p < 0.05 between basal vs androstenedione.

In isolated cells, the basal mRNA abundance for bcl-2 in EEC was 0.47 ± 0.07 AU and for bax 0.39 ± 0.25 AU and in ESC, basal expression of bcl-2 mRNA was 0.87 ± 0.16 AU and 1.09 ± 0.10 AU for bax. Androstenedione induced a decrease of 58% of bcl-2 mRNA expression (p < 0.05) and a 30% increase of bax mRNA (p < 0.05) in EEC (Figure 2); therefore, the ratio bcl-2/bax was significantly lower compared to the basal condition (p < 0.05). In ESC, no significant differences on mRNA expression for bcl-2 and bax were found between the basal and androgen-treated conditions. No important changes in mRNAs expression were observed when hydroxyflutamide was added to both cell culture systems.

### Effect of androstenedione on messenger RNA abundance of steroid receptors

In the endometrial explant cultures, the abundance of mRNA for ERα was similar between the basal condition and the tissue treated with androstenedione (0.44 ± 0.12 AU; 0.41 ± 0.07 AU, respectively). In contrast, androstenedione treatment induced a decrease in ERβ mRNA abundance (basal: 0.91 ± 0.11 AU ; treated: 0.75 ± 0.08 AU; p < 0.05). However, the ratio between the level of ER isoforms did not change. No significant modifications were observed when hydroxyflutamide was added to the cultures.

On the other hand, basal expression of ERα mRNA was 0.31 ± 0.05 AU and 0.39 ± 0.08 AU for ERβ in EEC; in ESC was 0.82 ± 0.23 AU for ERα and 0.86 ± 0.28 AU for ERβ.

**Figure 3:**
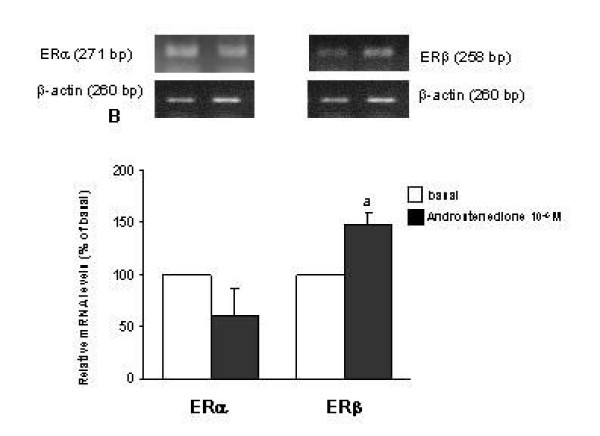
Polymerase chain reaction (PCR) amplification from reverse-transcribed cDNA from endometrial epithelial cells (EEC) under the stimulation with androstenedione 10^-6 ^M, using primers for ERα, ERβ and β-actin. Results represent six experiments performed in duplicate. Normalized yield for ERα and ERβ PCR fragments relative to β-actin. PCR products from different experiments are shown as percentage respect to basal. The values are expressed as mean ± SEM. ^a ^P < 0.05 between basal vs androstenedione.

Androstenedione tended to modify mRNA abundance of ERα in EEC and ESC, a 30% and 25% diminution was obtained, respectively (p = 0.07). In EEC, gene expression of ERβ increased 48% with androstenedione (0.39 ± 0.08 AU vs 0.56 ± 0.18, p < 0.05) (Figure 3), with no modification in ESC. Therefore, the ratio between the expression of ER isoforms decreased 70% in EEC. Hydroxyflutamide reverted the effect of androstenedione on gene expression of ERβ and on the ratio ERα/ERβ.

## Discussion

The present investigation represents an interesting approach that associates the potential effect of androgens on endometrial cell survival. By means of two *in vitro *models, we could observe that androstenedione can modulate the proliferation and apoptosis of the stromal compartment and modify the mRNA abundance of proteins related to apoptosis and β-isoform of ER in EEC.

Thereby, in explant cultures, androstenedione stimulated cell proliferation in stroma. One possible explanation to this finding may be related to the fact that androgens can induce the expression of the receptor of epidermal growth factor in the stromal compartment, as reported previously [25]. In turn, the increase in the proliferation rate may occur through an indirect effect of the growth factor on stromal cells.

Also, in the tissue explants model we observed that androstenedione induced a diminution in the apoptosis degree; although, the abundance of bcl-2 gene decreased. This observation may be in contrast to the mechanism that regulates apoptosis in cells; however, the ratio bcl-2/bax did not change and, concomitantly, we did not observe important differences in the expression of caspase-3. This is a relevant point because according to previous reports the control of cell death is principally associated with an unbalance in the expression of proteins related to the bcl-2 family, mainly bcl-2 and bax [17,18]. These findings may suggest that alternative apoptotic pathways can be also operating in the endometrial cells.

On the other hand, it is well known that in endometrial tissue, estrogen actions mediated through their own receptors have been related to cell survival and progression of proliferation. Furthermore, during the menstrual cycle, it has been demonstrated the presence of the isoforms α and β of ER in endometrial tissue [26,27]. Moreover, it has been postulated that the expression of ERα may be associated to bcl-2 gene expression [28]. Studies in other reproductive tissues have also suggested that ERβ could be involved in the inhibition of cell proliferation [29,30]. Therefore, it acquires great relevance the relationship between α and β isoforms of ER, considering that both isoforms could have an antagonistic action ligand-dependent, in accordance to the relative expression of each isoform in different tissues [16,31]. In the tissue explant model used in the present study, in contrast to a previous report [8], we were unable to demonstrate an increase of ERα. Nevertheless, we observed a decrease of the β-isoform of ER under the effect of androstenedione, which could be associated to the increase of stromal cell proliferation detected in the explants. Numerous clinical and *in vitro *studies have suggested that the imbalanced of ERα/ERβ is a common feature and could be critical in the progression of estrogen-dependent tumors. It seems that ERβ is an important modulator of the mitogenic action estrogen and it confers protection against the ERα hyperproliferation [32]. Moreover, in prostate carcinoma cells the expression of ERβ has been associated with triggers of apoptotic pathway, similar to observed in models of ovarian cancer cells [33,34]. Therefore, the observations in our models open an important field in a possible relationship between androgens effects with control of ERβ-mediated cell proliferation

When isolated cells were evaluated, some differences were obtained in the parameters studied compared to the explant cultures, which highlight the importance of the relationship between the different cell compartments in the regulation of the cell survival [35].

In ESC, androstenedione stimulated cell proliferation with no changes in the apoptosis degree nor on the expression of the genes bcl-2 and bax. This observation suggests that ESC exhibit an independent capacity to respond to androstenedione, whose action may be mediated by the androgen receptor. The latter is based mainly on the effect of the competitive inhibitor of the androgen receptor, hydroxyflutamide, which reverts the effect of androstenedione in the two models.

In EEC, androstenedione induced a diminution in the ratio between the mRNA abundance of bcl-2 and bax without an evident increase in the apoptosis degree. Previous studies in other models, such as breast cancer cell lines, have demonstrated that androgens can induce a decrease on the expression of bcl-2 and also an atrophy of the mammary ephitelium [36]. The present observations suggest that the mechanisms of control of cell death in EEC are different from those of ESC, indicating that ESC may be responsible in providing molecular and physical interactions that can inhibit the early changes in the balance of apoptosis control genes in EEC.

In contrast to our results, a previous study showed that in EEC cultures, androstenedione produces a fall in cell proliferation [14]. In fact, we did not observe this phenomenon, most likely due to technical differences in which the treatment was performed for 24 h in the present study with the aim to evaluate the early expression of genes related to apoptosis.

Moreover, in EEC we observed that androstenedione up-regulated the ERβ expression and hence, a decrease in ER/ERβ ratio was obtained. The meaning of this effect on cell survival is difficult to evaluate in our experimental model, since no estradiol was added to the culture system. Furthermore, it is unlikely that androstenedione may produce estrogen by P_450 _aromatase activity, because normal endometrial cells present a very low expression of this enzyme, as previously demonstrated [37]. However, in EEC the decrease in ERα/ERβ may be related to an unbalance of the bcl-2/bax ratio although this hypothesis needs further studies.

Taking together our results, we can speculate that in the presence of androgens the regulatory mechanisms of cell survival in endometrial cells would be associated to the interaction capacity elicited by the different cellular components of the tissue. In fact, we postulate that androgens induce an increase of proliferation in stroma, probably related to growth factors and that these signals interact with epithelial cells, promoting an inhibition on the expression of genes related to cell death. In isolated cells, the mechanisms that allow these interactions between cell compartments are lost and the cells act according to their proper feature.

Our findings on the effect of androstendione on cell proliferation and apoptosis of ESC allows us to suggest a potential regulatory action of androgen in the physiology of the endometrium and its implications in the genesis of endometrial pathologies frequently found in women with hyperandrogenism. Even more, we have observed that endometrial androstenedione concentration in women with PCOS are three times higher than in normal women during the proliferative phase [unpublished data). Therefore, these in vitro models are important approaches to understand the potential role of androstenedione in the PCOS endometria. It is well established that women with PCOS exhibit a high prevalence of hyperplasia and endometrial cancer, which is associated with disturbances in the regulation of cell survival [4-6]. According to our results, androgens may be involved in these endometrial alterations. However, we cannot ruled out the possible action of hyperinsulinaemia, common feature observed in PCOS women, which has been associated to increase the potential for neoplastic change through theirs effects on sex hormone binding-globulin (SHBG), insulin-like growth factor-1 and estrogen concentrations [6,38].

## Conclusions

In summary, our results indicate that androstenedione may modulate cell survival, the expression of β-ER isoforms and proteins related to apoptosis. These observations are closely related to the control of endometrial cell function and may help to understand the possible effect of the hyperandrogemia on endometrial tissue.

## Authors' contributions

MM conceived and designed the study, carried out the experimental protocols, and drafted the manuscript. SQ, MC and KB performed the RT-PCR and scored of immunohistochemistry, MA carried out the score of immunohistochemistry studies. CJ reviewed and supported in the drafting the manuscript and MV conceived the study as principal investigator and participated in drafting the manuscript.
